# Pain experiences of adults with osteogenesis imperfecta: An integrative review

**DOI:** 10.1080/24740527.2017.1422115

**Published:** 2018-01-30

**Authors:** Tracy Nghiem, Khadidja Chougui, Alisha Michalovic, Chitra Lalloo, Jennifer Stinson, Marie-Elaine Lafrance, Telma Palomo, Noémi Dahan-Oliel, Argerie Tsimicalis

**Affiliations:** a Ingram School of Nursing, McGill University, Montreal, Quebec, Canada; b Shriners Hospitals for Children-Canada, Montreal, Quebec, Canada; c The Hospital for Sick Children, Toronto, Ontario, Canada; d Lawrence S. Bloomberg Faculty of Nursing, University of Toronto, Toronto, Ontario, Canada; e Bone and Mineral Unit, Division of Endocrinology, Universidade Federal de São Paulo, Sao Paulo, Brazil; f School of Physical and Occupational Therapy, McGill University, Montreal, Quebec, Canada

**Keywords:** pain management, pain perception, pain measurement, brittle bone, Lobstein, knowledge synthesis

## Abstract

**Background**: Pain is a common symptom of osteogenesis imperfecta (OI) among children and adolescents. However, little is currently known of the pain experiences of adults with OI.

**Aims**: The aims of this study were to critically appraise the studies assessing OI pain, to synthesize the pain experiences of adults with OI, and to compare the adult OI pain experiences to childhood.

**Methods**: An integrative review was conducted. Five electronic bibliographic databases were searched. Published quantitative, qualitative, and/or mixed-method studies assessing pain in adults with OI were screened, reviewed, and appraised. Descriptive statistics were used to calculate quality scores, summarize sample characteristics, and synthesize findings. Extracted pain data were analyzed using constant comparison and consolidated into meaningful themes.

**Results**: From the 832 titles identified, 14 studies including seven case reports met the inclusion criteria. Study appraisal scores ranged from low to moderate using the Quality Assessment Tool and the Case Report Checklist. The majority of studies assessed pain as a secondary outcome (71.4%) using well-established tools (64.2%). Adults with OI experience pain of mild to moderate intensity, which may interfere with completion of daily activities. Two themes emerged from analysis of the data: mild chronic pain persists despite surgical, pharmacological, or nonpharmacological interventions and past fractures and structural deformities may trigger onset of chronic pain in adulthood.

**Conclusion**: Limited attention has been given to exploring the pain experience of adults diagnosed with OI. Pain is a long-term symptom of OI requiring further in-depth investigation to better understand and manage pain in adults with OI.

## RÉSUMÉ

**Contexte**: La douleur est un symptôme commun de l’ostéogenèse imparfaite (OI) chez les enfants et les adolescents. Toutefois, on sait actuellement peu de choses au sujet de la douleur ressentie par les adultes atteints d’OI.

**But**: Effectuer une appréciation critique des études évaluant la douleur occasionnée par l’OI, faire la synthèse de l’information sur la douleur ressentie par les adultes atteints d’OI et comparer la douleur ressentie à l’âge adulte à celle ressentie pendant l’enfance.

**Méthodes**: Un examen par intégration a été mené. Des recherches ont été effectuées dans cinq bases de données bibliographiques électroniques. Les études quantitatives, qualitatives ou mixtes publiées qui évaluaient la douleur chez les adultes atteints d’OI ont été sélectionnées, examinées et évaluées. Des statistiques descriptives ont été utilisées pour calculer leur score de qualité, résumer les caractéristiques de leur échantillon et synthétiser leurs conclusions. Les données sur la douleur qui avaient été extraites ont été analysées à l’aide de la méthode de la comparaison constante et regroupées en thèmes significatifs.

**Résultats**: Parmi les 832 titres recensés, 14 études comprenant sept rapports de cas répondaient aux critères d’inclusion. Au moment de les évaluer, les études ont obtenu un score de qualité allant de faible à modéré en utilisant l’Outil d’évaluation de la qualité et la Liste de vérification pour les études de cas. La majorité des études évaluaient la douleur en tant que résultat secondaire (71,4 %) à l’aide d’outils bien établis (64,2 %). Les adultes attents d’OI ressentent une douleur d’une intensité allant de légère à modérée, qui peut perturber leurs activités quotidiennes. Deux thèmes ont émergé de l’analyse des données : « La douleur chronique légère persiste malgré les inerventions chirurgicales, pharmacologiques ou non phramacologiques » et « les fractures passées et les malformations structurelles peuvent déclencher la douleur chronique à l’âge adulte ».

**Conclusion**: L’étude de la douleur ressentie par les adultes ayant reçu un diagnostic d’OI n’a reçu qu’une attention limitée. La douleur est un symptôme d’OI à long terme qui nécessite une investigation plus approfondie afin de mieux la comprendre et d’en assurer une meilleure prise en charge chez les adultes atteints d’OI.

## Introduction

Osteogenesis imperfecta (OI) is the most common heritable bone fragility disorder that affects approximately one in 10 000 individuals.^1^ There are currently five types of OI,^2,3^ where types I to IV are the most frequently diagnosed and encountered in the clinical setting.^3^ Regardless of the OI type, growth deficiencies, skeletal fragility, and deformities are the main observed clinical characteristics.^4^ As a consequence, frequent fractures, bone pain, and varying degrees of physical limitations are seen with individuals diagnosed with OI.^5,6^

**Figure 1.**
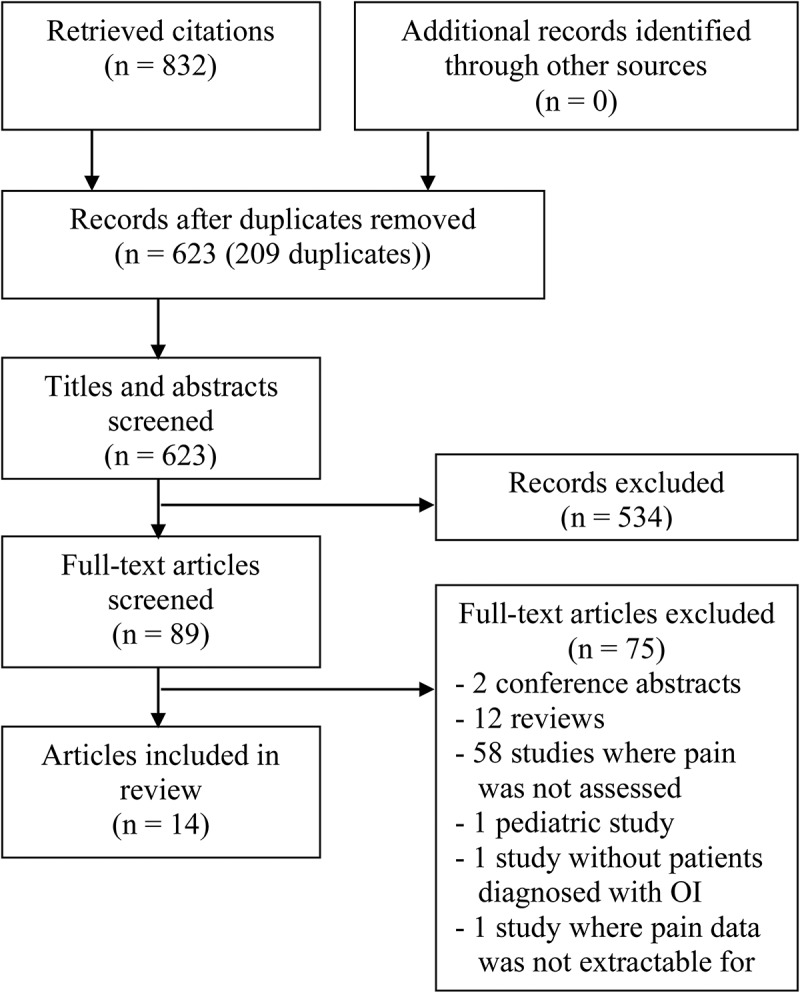
Flow diagram of study selection.

Pain is a complex, multidimensional subjective phenomenon.^7,8^ The pain dimensions that are commonly measured are (1) sensory (i.e., intensity, quality, location, duration), (2) affective (i.e., emotional unpleasantness), and (3) evaluative (i.e., interference with social or daily living functioning).^9,10^ Pain is also distinguished by its duration in time (i.e., acute versus chronic) requiring different treatment modalities. Pain that is provoked by a threat to the body (e.g., following a fracture) is referred to as acute pain and it is generally time limited.^11^ Pain that persists long after healing has occurred is referred to as chronic pain.^7^

A recent integrative review consisting of 19 studies sought to describe the pain experiences in young individuals with OI.^12^ Among children and adolescents with OI, acute and chronic pain is present and problematic. Most notably, chronic pain interferes with sleep, mobility, and participation in school and activities. The study also highlighted the paucity of research and methodological issues with assessing pain in this population. Although there are three well-known dimensions of pain, the data collected on pain experiences of children and adolescents with OI were mainly sensorial. The reviewers concluded that pain related to OI must be more comprehensively assessed to facilitate future pain management strategies for individuals diagnosed with OI.

As individuals with mild types of OI can expect a similar life span to the general population^13^ and individuals with more severe forms of OI may live past adolescence,^14^ greater efforts are needed to understand the pain experiences into adulthood. Currently, there are no existing reviews that examine the pain experienced by adults diagnosed with OI. Thus, the objectives of this integrative review were to (1) describe the pain experiences of adults with OI; (2) determine how adult OI pain is being assessed; (3) determine the methodological quality of OI pain studies; (4) compare and contrast the adult OI pain experiences to childhood; and (5) identify implications for research and practice.

## Methods

### Study design

An integrative design was chosen to systematically review, appraise, extract, and synthesize the data. An integrative review adheres to a rigorous process similar to a systematic review^15^ but permits the integration of quantitative, qualitative, and mixed-method study findings using descriptive statistics and constant comparison methods.^16^

### Information sources and search strategy

Studies selected for inclusion in the review were accessed through a search of CINAHL (1937–July 2016), EMBASE (1947–July 2016), Medline (1946–July 2016), PsycInfo (1987–July 2016), and Joanna Briggs Institute (1996–July 2016). The search strategy was developed in collaboration with a librarian scientist. The search terms included MeSH headings, subjects headings, text words, and/or keywords with or without truncations and explosions where applicable relevant to the following terms: “osteogenesis imperfecta,” “brittle bone*,” “Lobstein*,” “pain,” “pain management,” “pain perception,” “pain measurement,” “pain threshold,” and “nocicept*.” These terms were chosen to best reflect the conceptualization of pain.^8^ The date of the last search attempt was July 14, 2016. The full electronic search strategy for each database is available upon request (Supplemental Table 1). Lastly, reference lists from review papers and papers identified as appropriate were hand searched for any relevant additional studies. No attempt was made to locate unpublished materials or contact researchers for unpublished studies. The bibliographic software EndNote X7 was used to manage the collected citations.

**Table 1.** Study characteristics.

					Sample characteristics					
Author, year, country	Type of journal	Study design	Study purpose	Control group	Sample size	Age (years)	Gender	OI type	Pain assessment and tools used	Reported reliability and validity of pain tool in assessing pain
Balkefors et al., 2012, Sweden^21^	Physiotherapy	Prospective cross-sectional study	Describe physical activity, quality of and satisfaction with life, pain, joint mobility, and muscle function	Y (Swedish adult population data used as comparison)	29	Median: 41 Range: 21–71	18 F, 11 M	Types I and IV	SF-36 Pain drawing Use of descriptive words	No Yes No
Bradbury et al., 2012, Australia^20^	Orthopedic/endocrine	Prospective observational study	Investigate the effect of risedronate in adults with OI type I	N	33	Mean: 39 Range: 18–76	21 F, 11 M	Type I	VAS VRS (0–10)	No No
Chevrel et al., 2006, France^19^	Orthopedic/endocrine	Randomized, double-blind, placebo-controlled trial	Evaluate the effect of oral alendronate on bone mineral density	Y	64	Placebo: Mean: 37 ± SD 12 Treatment: Mean: 36 ± SD 12	25 F, 39 M	Type I (62), type IV (2)	VAS	No
Furstenburg et al., 2010, Germany^26^	*European Spine Journal*	Case report	Report the use of khyphosplasty	N/A	1	58	M	Type I	VAS	No
Hardernbrook and Lombardo, 2006, United States^30^	Neurosurgery	Case report	Report the use of khyphosplasty	N/A	1	25	M	Unknown (diagnosed with visit)	Physical examination VRS	N/A No
Iwamoto et al., 2003, Japan^28^	Orthopedic	Case report	Present a case of OI successfully treated with oral etidronate and alfacalcidol	N/A	1	36	M	Type I	Face Scale Score	No
Iwamoto et al., 2004, Japan^27^	Medicine	Case report	Present a case of OI successfully treated with aledronate	N/A	1	41	F	Type I	Face Scale Score	No
Khoury et al., 2008, Canada^31^	*Journal of Vascular Intervention Radiology*	Case report	Report the use of a modified transpedicular vertebroplasty approach for treatment of a vertebral body fracture	N/A	1	66	F	Type I	VAS	No
Kim et al., 2013, Korea^32^	Medicine	Case report	Report the use of an epidural and spinal nerve block to treat chronic low back pain	N/A	1	42	F	Unknown	Physical examination VAS	N/A No
McAllion and Paterson, 2002, UK^22^	Obstetrics and gynecology	Qualitative, retrospective cross-sectional study	Determine the likelihood of pain and other musculoskeletal problems in pregnancy	N	100	Age at time of participation, range: 18–50 Age at delivery (or abortion) ranged from 15 to 42, mean: 25.9	100 F	Types I, III, IV	Created survey	No
McKiernan, 2005, United States^23^	Orthopedic/endocrine	Qualitative cross-sectional study	Characterize the musculoskeletal manifestations and resulting impairments	N	111	Range: 20–70 Men: Mean: 42.1 Women: Mean: 40.2	78 F, 33 M	Mild OI only	Created survey	No
Nicolaou et al., 1994, UK^24^	Orthopedics/endocrine	Qualitative, retrospective cohort study	Evaluate the long-term outcomes and complications of the Sheffield rod system	Y (UK population data used as comparison)	22	Mean: 24.7 Range: 18–36 Median: 23.5 At time of rod insertion 2.0 years–13.3 years, mean: 5.83	13 F, 9 M	Types I (7), III (10), and IV (5)	SF-36	No
Papagelopoulos and Morrey, 1993, United States^25^	Orthopedics/endocrine	Qualitative retrospective cohort study	Describe the management of severe osteoarthrosis of the hip or knee with recipients of a joint replacement	N	6	Age at the time of replacement: Hip Mean: 61 Range: 60–62 Knee Mean: 50 Range: 46–54	4 F, 2 M	Types IA (1), IIA (1), III (2), IVA (1), and IVB (1)	Harris Hip Score Knee Society Score	No No
Rami et al., 2001, United States^29^	*Skeletal Radiology*	Case report	Report a case of percutaenous vertebroplasty in a patient with vertebral body compression fracture secondary to OI	N/A	1	57	M	Type I	Physical examination VRS	N/A No

OI = osteogenesis imperfecta; SF-36 = 36-Item Short-Form Health Survey; VAS = Visual Analogue Scale; VRS = Verbal Rating Scale.

### Study selection

Two reviewers independently screened identified titles and abstracts, and full-text articles were read independently by the same two reviewers to examine for relevance according to the eligibility criteria. Any discrepancies arising during this process were resolved by discussion with members of the research team until consensus was achieved. These discussions continued throughout the entire study.

### Eligibility criteria

#### Types of participants

Studies including adults (18 years and older) diagnosed with any OI type were included to obtain an in-depth portrayal of OI pain in adulthood.

#### Types of outcomes

Any study design assessing OI pain as a primary or secondary outcome was included. Assessments on any pain dimension (i.e., sensory, affective, and/or evaluative) using any type of pain assessment method (e.g., physiological, self-report, and behavioral) were included.

#### Types of studies

All quantitative, qualitative, and mixed-methods studies published in peer-reviewed journals were included. Case reports were also included to provide further pain insight. There was no minimum threshold for quality. No restrictions were placed on the basis of country or date of publication; however, language was restricted to English, French, and Spanish publications due to language capacity of the research team.

### Data evaluation

Eligible studies were appraised by two independent reviewers using the Quality Assessment Tool or Case Report (CARE) Checklist.^17,18^ The Quality Assessment Tool was chosen a priori because the tool permits appraisal of studies across a range of designs (i.e., quantitative, qualitative, or mixed method) and would allow the findings to be compared to the review on pain in children and adolescents with OI.^12^ A number from 0 to 3 was allocated to each study based on a number of criteria, such as evidence of sample size calculation, description of the procedure for data collection, and provision of detailed recruitment data. The final quality score was calculated as a percentage of the highest possible score. The CARE checklist is a reporting guideline for case reports and was adapted by the team to appraise the included case studies. Each item on the checklist was scored as 0 (not met) or 1 (met). The quality score was calculated as a percentage of the highest possible score. Appraisal scores range from 0% (lowest score) to 100% (highest score).

### Data extraction

To summarize the study characteristics and pain findings, data were extracted into a table created in Microsoft Word by one reviewer and verified by a second. Extracted data included author, year of publication, type of journal, purpose, study design, sample characteristics (e.g., size, age range, sex, OI type), data collection methods, pain as a primary or secondary outcome, inclusion of a definition of pain, type of pain report, time points of pain assessment, type of pain (i.e., temporality, modality), pain assessment method, sensory characteristics of pain (i.e., intensity, duration and frequency, location, quality), emotional aspects of pain, impact of pain, and other pain findings.

### Data analysis

Descriptive statistics were used to generate the flow diagram (Figure 1), calculate quality scores, summarize sample characteristics, and synthesize findings (where appropriate). Extracted pain data were analyzed together using constant comparison. Each extracted categorical item was compared to another, grouping similar data together.^16^ These groupings were subsequently consolidated into meaningful themes describing patterns across the data to characterize the pain experiences of adults with OI.

## Results

### Selection strategy and methodological quality

A total of 832 articles were imported into Endnote X7; after removing duplicates, 623 articles remained (Figure 1). The titles and abstracts were then reviewed and 89 articles were retained. A total of 38 titles and abstracts were excluded due to the full text of the study being in a language other than English, French, or Spanish. The full texts of 51 English, French, or Spanish studies were reviewed by two reviewers to ensure that the studies met the inclusion criteria. Of these studies, seven case reports and seven quantitative studies were included for methodological appraisal, resulting in varying degrees of quality (Supplemental Table 2). The quality scores of the seven case reports range from 40% to 66.7%, with a mean of 56.7% ± 9.8 and median of 60%. The quality of the seven quantitative studies range from 25.0% to 52.4%, with a mean of 39.1% ± 9.4 and median of 36.9%. All 14 studies were reviewed.

**Table 2.** Pain tools used by reviewed studies.

Pain tool	Self-reported pain	Description	Pain dimensions	Recommended by IMMPACT guidelines^33^
Face Scale Score	No	Pain evaluated by assessing the mood of the patient according to the Face Scale Score. Scores are arranged in decreasing order of mood and numbered from 1 to 10, with 1 representing the most positive mood and 10 representing the most negative mood.^28,a^	Sensory–Intensity	No
Harris Hip Score	Yes	Evaluation of hip through pain, function, range of motion, and absence of deformity. Pain is scored along with activity interference.^50^	Sensory–Intensity Cognitive	No
Knee Society Score	Yes	Evaluation of knee through evaluating knee score through range of motion and flexion/extension as well as self-reported function score through pain and ability to walk or use stairs.^51^	Sensory–Intensity Cognitive	No
Pain descriptive word list	Yes	From a list of descriptive words, patient picks word that best describes his or her pain.^21^	Sensory–Quality	No
Pain drawing	Yes	Patients report pain by coloring a body chart to locate their pain and indicate extent of pain.^52^	Sensory–Location	Yes
SF-36	Yes	Contains 36 questions with fixed answers on different raw scales. The questions cover eight domains: physical functioning, role physical, bodily pain, general health, vitality, social functioning, and role emotional and mental health. Scores vary between 0 and 100. A high score indicates high quality of life.^53^	Sensory–Intensity Cognitive	Yes
Survey by McAllion	Yes	The following information was collected for each pregnancy: on date and mode of delivery, loss of height (if any), fractures during pregnancy, back pain (onset and duration), deafness (if any), whether bone densitometry was carried out, and any other complications.^22^	Sensory–Intensity	No
Survey by McKiernan	Yes	A 32-question survey was constructed to characterize the nature and severity of musculoskeletal manifestations to estimate their degree of impairment. The survey emphasized those musculoskeletal issues that the existing scientific literature reports to be of concern to this population, specifically, fracture, arthritis, scoliosis, back pain, joint hypermobility, tendonopathy, and complex regional pain syndrome.^23^	Sensory–Intensity	No
VAS	Yes	Patient marks pain on a premeasured line. The line may differ in units of measurement and length.^54^	Sensory–Intensity	Yes
VRS	Yes	Patient verbally reports pain using a numerical scale. In most cases the scale ranges from 0 (*no pain*) to 10 (*most severe pain*).^55^	Sensory–Intensity	Yes

^a^Case report.

SF-36 = 36-Item Short-Form Health Survey; VAS = Visual Analogue Scale; VRS = Verbal Rating Scale.

### Study characteristics

The characteristics of the 14 studies are summarized in Table 1. The seven quantitative studies included two experimental designs, with one being a randomized controlled trial^19^ and the other a nonrandomized and noncontrolled trial,^20^ as well as five cross-sectional designs,^21–25^ three of which relied on retrospective data collection methods such as chart reviews.^22,24,25^ There were no qualitative or mixed-methods designs and no study included participants to help design, interpret, or disseminate the research. Studies were conducted in nine different countries and subsequently published in English in medical (*n* = 2), neurosurgery (*n* = 1), orthopedic/endocrine (*n* = 6), vascular surgery (*n* = 1), physiotherapy (*n* = 1), skeletal radiology (*n* = 1), spinal surgery (*n* = 1), or obstetrics and gynecology (*n* = 1) journals. There were no studies published in pain journals. Studies were published between 1993 to 2013.

#### Sample characteristics

In total, 371 adults with OI participated in the studies, including 262 females (70.6%) and 109 males (29.4%) with Type I (*n* = 101), II (*n* = 1), III (*n* = 12), IV (*n* = 9), “mild” (*n* = 111), “unknown” (*n* = 2), or unspecified OI (*n* = 129). The age of participants ranged from 18 to 76 years. In the seven case reports, age ranged from 25 to 66 years, 57% of participants were male and 71% were diagnosed with OI Type I. Excluding case reports, sample sizes ranged from six^25^ to 111^23^ participants with a median of 32. Participation rate ranged from 84.4% to 100%.

A history of at least one or more fractures was noted in up to 96% of participants (*n* = 29) in one study.^21^ In a second study (*n* = 33 males, *n* = 78 females), the total number of self-reported lifetime fractures reported was 1190 for males and 2220 for females, with the individual average of sustained lifetime fractures being 31.^23^ Within this same study, up to 28% of the total number of fractures between both males and females occurring after 18 years of age were located most often in the spine (47.3%), followed by lower extremities (30.2%) and then by upper extremities (21.4%).^23^ Major fracture incidence in a 2-year study period with 27 participants with OI Type I was 0.18 and 0.15 excluding postmenopausal women.^20^ In four of the seven (57.1%) case reports, participants had a history of fractures,^26–29^ and in six of the seven (85.7%) case reports, spinal fractures were present in the patients upon their visit to the clinic.^26,27,29–32^ In five of the seven quantitative studies, 23% to 79% of participants had spinal deformities (khyphosis or scoliosis).^19–21,23,25^ Similarly, khyphosis or scoliosis was noted in two of the seven case reports (28.5%).^26,27^

#### Pain assessments

##### Study outcomes

In the seven quantitative studies, pain was measured as a primary (28.6%)^22,25^ or secondary (71.4%)^19–21,23,24^ outcome with pain assessments conducted (1) during or after specific events (71.4%), including bisphosphonate treatment,^19,20^ surgery,^24,25^ and childbirth,^22^ or (2) to describe impact of pain on functioning and quality of life (QoL; 28.6%).^21,23^ The majority of studies did not specify what type of pain was being assessed with the exception of one study that assessed chronic pain.^21^ All participants self-reported their pain, except in two case studies where an observational pain scale (the Face Scale Score) was used.^27,28^ In these two case studies, there was no rationale given for evaluating pain through observation rather than self-report. All seven case reports reported that pain was the main reason for consultation; in six of these cases,^26–28,30–32^ chronic pain was present. Only one case study observed acute pain from a fracture related to an automobile tire change; the patient had no previous history of pain.^29^

##### Pain assessment tool(s) used

Ten different tools were used to assess pain in the hospital setting or over the telephone (Table 2). Two studies used an investigator-created survey for the purpose of their study,^22,23^ and eight studies (64.2%) used reliable and valid tools for assessing either of the three dimensions of pain as recommended by the IMMPACT guidelines.^33^ The Harris Hip Score, the Knee Society Score, the 36-Item Short-Form Health Survey (SF-36), and the self-created survey by McKiernan et al.^23^ were not intended to be used to assess pain as a primary outcome but rather have pain items imbedded in the tools to help guide the assessment of hip or knee function, QoL, and musculoskeletal functioning.

##### Time points of assessment

Thirteen studies assessed current pain at the time of the study. Pain reporting bias was only evident in one study that relied on recall of pain during pregnancy 4 to 45 years after the event.^22^ Two quantitative studies and four case studies assessed pain at baseline and at regular 4- to 6-month intervals after intervention (bisphosphonate treatment, khyphoplasty, vertebroplasty, epidural and spinal nerve block treatment) for 9 months to 3 years.^19,20,27,28,30,32^ Three studies assessed pain at baseline and once postoperatively (khyphoplasty and vertebroplasty),^26,29,31^ two studies evaluated pain only once postoperatively,^24,25^ and two studies provided a one-time cross-sectional assessment of pain.^21,23^

##### Dimensions of pain

The sensory dimension was assessed in all 14 studies, including intensity, quality, and location. Baseline mean pain intensity before any intervention (i.e., bisphosphonate or epidural or spinal block treatment, khyphoplasty, vertebroplasty) took place ranged from 2.7 to 10 in studies using the Visual Analogue Scale (VAS),^19,20,26,31,32^ 4.0 to 5.0 in studies using the Verbal Rating Scale (VRS),^20,29^ and 7.0 on 10.0 in studies using the Face Scale Score.^27,28^ Mean pain intensity at the latest visit post-bisphosphonate or epidural or spinal block treatment, khyphoplasty, or vertebroplasty ranged from 2.0 to 3.0 in studies using the VAS,^19,20,26,31,32^ 0 to 3.0 in studies using the VRS,^20,29^ and 7.0 on 10.0 in two case studies using the Face Scale Score.^27,28^ Using the Harris Hip Score and Knee Society Score, pain intensity ranged from no pain to slight pain after hip or knee replacement.^25^ In a study with 100 pregnant women with OI, 40% recalled experiencing mild levels of pain, 36% recalled experiencing moderate levels of pain, and 24% recalled experiencing severe levels of pain.^22^ In the two studies that used the SF-36, mean bodily pain ranged from 62 to 65 out of 100, indicating mild to moderate pain intensity.^21,24^ Compared to data collected from the general population of Sweden and the United Kingdom (mean bodily pain score 84/100 and 81/100, respectively), the SF-36 scores of individuals with OI were significantly lower (*P* < 0.001, in both cases). The quality of the pain was only described in one study; participants with OI described their pain as “annoying” or “discomforting.”^24^ In regards to location of pain, back pain was most commonly reported, with 66% to 76% of participants in two studies having back pain (total *n* = 34).^21^,^24^ In a third study, back pain had the highest composite score (a product of intensity and frequency) among other body parts, such as knees, hip, ankles, shoulder, elbows, and hands.^23^ In addition, all seven participants in the case studies presented with back pain.^26–32^ One study only looked at back pain during pregnancy,^22^ and one study examined knee or hip pain only.^25^

The evaluative impact dimension was assessed by five studies through exploring pain interference with activity.^21–25^ In one study using their own designed survey, three quarters of participants reported having back pain (*n* = 58, 52%) and had some degree of unspecified impairment due to the pain, resulting in the need for assistance with personal tasks.^23^ This was also the case in another study, where 68% (*n* = 15) of the participants reported that back pain led to difficulty with completing daily activities.^24^ In a third study, moderate correlations between bodily pain score and the ability to climb stairs (*r* = 0.69), go for walk (*r* = 0.60), and sitting for long periods of time (*r* = 0.59) were reported.^21^ In addition, severe pain was described to affect women’s ability to lift or walk during pregnancy (24% of pregnancies), and five women with severe back pain required traction or bed rest for 3 to 10 weeks.^22^ Though not assessed through a tool, two case reports also described their participants having difficulty mobilizing due to pain.^26,30^ Conversely, in a single study using the Harris Hip Score and Knee Society Score, the majority of participants (*n* = 5, 83%) had no compromise in their range of motion at the knee or hip joints after having a surgical intervention.^25^

No studies assessed the affect dimension of pain.

## Identified themes

### Mild chronic pain persists despite surgical, pharmacological, or nonpharmacological interventions

The majority of participants experienced pain at the time of study assessment. In six of the seven case reports, adults with OI had been experiencing chronic pain for several months. In three case reports, patients reported that conservative pharmacological treatment with analgesics, such as tramadol and nonpharmacological approaches, such as bracing, and/or modifying activity level provided very little pain relief.^26,29,30^ In two other cases, patients were not receiving any treatment for their chronic pain. After undergoing various interventions including bisphosphonate therapy,^19,20,27,28^ Sheiffield rod placements,^24^ vertebroplasty,^29,31^ kyphoplasty,^26,30^ knee or hip replacement,^25^ or pain management with an epidural and spinal block,^32^ participants still reported mild residual pain at least 3 years later. None of the studies reported whether participants continued using pharmacological and/or nonpharmacological approaches for pain relief after receiving these various interventions.

### Past fractures and structural deformities may trigger onset of chronic pain in adulthood

Across all studies, chronic back pain was the most commonly reported among adults with OI, with participants having notable back deformities or vertebral compression fractures. Three case reports and one quantitative study included a detailed account of a history of fractures with the incidence highest in infancy or adolescence and the occurrence of fractures persisting throughout adulthood.^23,27–29^ Six of the seven case reports concluded that the back pain was not linked to injury but rather to structural deformities and vertebral compression. Likewise, two of the quantitative studies reported that khyphosis and scoliosis were common among their participants with back pain.^21,23^ In addition, pregnant participants with OI with severe pain were all found to have clinical or radiological evidence of vertebral compressions; those with mild or moderate pain had no clear cause that could be identified.^22^

## Discussion

This integrative review appraised, reviewed, and synthesized the findings of 14 studies published between 1993 and 2013. Findings revealed that OI pain is present, problematic, and persists into adulthood, with the majority of adults experiencing mild chronic pain despite surgical, pharmacological, or nonpharmacological interventions. OI pain in adults is primarily located in the back area and may be triggered from previous fractures and structural deformities. Collectively, these findings enrich our understanding of the pain experienced by adults with OI, allowing us to compare to the synthesized literature on children with OI, comment upon the methods of pain assessment and the low to moderate quality of research on OI pain, as well as identify implications for research and practice.

### Findings compared to children with OI

Many similarities exist between the pain experiences of children and adults with OI. Like children with OI, adults experience pain in a variety of locations ranging from mild to severe intensity.^12^ In addition, pain has an impact on the daily activities in both children and adults with OI,^12^ which may negatively influence aspects of their QoL, especially the physical domain.^34^ Comparable to the childhood literature, there is insufficient evidence to describe the emotional impact of pain in adults with OI. Among children with OI, preliminary evidence derived from one study suggests that pain is associated with negative emotions described as “awful,” “frightening,” and “sickening.”^35^ These negative emotions may contribute to children having an extensive fear of fractures and of situations that may cause a fracture, limiting the number of people who may handle them, and suppressing their expressions of pain.^36^ Furthermore, children and adults with OI recognize the influence of their emotions on others and often seek to lessen their caregivers’ frustrations due to their inability to alleviate their pain.^36,37^

Only one case study in this review specified studying acute fracture pain. Similar to the child literature on OI pain, only one study specifically assessed for acute fracture pain.^12,35^ It is difficult to determine whether acute fracture pain in adulthood is similar to or different from experiences from childhood and to compare acute fracture pain to chronic nonfracture pain due to the lack of evidence. In a study on fracture rates and sites of individuals with OI, it was reported that the fracture incidence rate for children and adolescents was higher compared to persons aged 20 years and over.^38^ Although this indicates that acute pain experiences due to fractures in adults may be less than those in children, fractures may still occur triggering an acute pain experience and may lead to a chronic pain response.

Finally, pain is not well assessed in both children and adults with OI, and there are few reliable prospective studies measuring pain as a primary endpoint.

### Methods of pain assessment

Gaps in the pain data can be attributed to the methodological limitations in assessing pain among adults with OI. Across the 14 studies, varying tools were used to assess pain, which included tools without established reliability or validity (see Table 2). These tools were also used to assess pain in the hospital setting or over the telephone, capturing pain at a single point in time or at monthly intervals. Pain was often assessed before, during, and/or immediately after an event, such as bisphosphonate therapy, surgery, or pregnancy, and did not account for pain assessments outside these events. Of the 10 tools used across the 14 studies reviewed, seven were unidimensional in nature, assessing only the sensory or evaluative domain of pain. Several reasons may underline the brief pain assessment that is apparent in the studies, such as (1) the need for a quick and easy use of unidimensional pain tools^39,40^; (2) a nonpain target audience (because no study was published in a pain-related journal); and (3) pain was measured as a secondary outcome, which may explain why in certain studies, pain-specific tools were not used and pain assessments were not multidimensional, especially for chronic pain. These methodological issues in assessing pain in adults with OI are shared with the research literature on pain in children with OI.^12^ Hence, an in-depth understanding of the pain experiences of individuals with OI is warranted.

### Quality of pain research

The seven quantitative studies included in the review were of low to moderate quality. This can be attributed to common missing criteria across the studies, which included a rationale for choice of data collection methods, reporting of the reliability and validity of collection methods, an explanation for the sample size, and providing research questions. The seven case studies were of moderate quality. A number of case reports omitted the patient’s perspective, consideration of other diagnoses upon the patient’s initial presentation, inclusion of other possible interventions and a rationale for why they decided to choose one intervention over another, and a description of the strengths and limitations of the case report. A low to moderate quality was also reported in the literature on pain experiences of children with OI.^12^

The study sample sizes of the quantitative studies were small, and of the 125 participants in which OI type was identified, 101 (81%) were of OI Type I. Of the seven case reports, five patients (71%) were of OI Type I. This makes it difficult to generalize the results to all adults with OI and adults with more severe types of OI specifically. In addition, none of the studies include participants diagnosed with OI Type V and above. The overrepresentation of mild types of OI (e.g., Type I) is a recurring limitation in the OI literature due to the rarity of the disorder and certain OI types, as well as the short life span of individuals with severe types of OI.^14,41^

### Recommendations

Further attention should be given to understanding the pain experiences of adults with OI knowing that chronic pain is persistent among this population. Pain is not only unpleasant but is also disabling. In a recent review, pain had the potential to negatively impact QoL, either directly or indirectly, through limiting physical functioning and community participation of individuals with OI.^34^ In fact, the QoL of individuals with OI is lower than that of the general population.^34^ By further exploring how adults with OI experience pain, we would be more apt to develop appropriate interventions to properly manage it and, hence, improve their QoL.

Pain experiences can potentially fluctuate from day to day and vary depending on events and underlying conditions and between age groups as well as OI types among individuals with OI. Prospective longitudinal studies are known to be advantageous for establishing sequences of events and identifying patterns over time and are needed to determine how acute and chronic pain experiences may vary over time, age, OI type, and events (e.g., fractures, medical interventions) with the use of multidimensional measures in varying settings in real time.^42^ Similar to other chronic pain conditions (e.g., arthritis and sickle cell disease), pain needs to be measured using real-time data capture methods such as pain diaries that can assess different dimensions of pain across time, including the poorly documented evaluative and affective domains of pain.^43–45^ Methods to capture real-time data may also be used to track acute pain along with chronic pain.^46^ The greatest drawbacks to using the pain diary and a longitudinal study design is recall bias, compliance, and completion^46^; however, this may not be an issue with the OI population who actively participate in research, as noted in the 14 studies reviewed, and with the introduction of electronic diaries to assess pain.^47^

The assessment of pain in adults with OI, which encompass the three domains of pain, should be guided by the IMMPACT guidelines for research and clinical purposes. The IMMPACT guidelines suggest that there are six core outcome domains to be included in assessing chronic pain: (1) pain intensity, (2) physical functioning, (3) emotional functioning, (4) participant ratings of global improvement, (5) symptoms and adverse events, and (6) participant disposition.^48^ For acute pain, the PedIMMPACT has set guidelines for outcome domains in children, but these can be used to assess pain in adults as well.^49^ These include (1) pain intensity, (2) global judgment of satisfaction with treatment, (3) symptoms and adverse events, (4) physical recovery, (5) emotional response, and (6) economic factors. In addition, certain outcome measures and assessment tools were recommended for each domain.^33^ Following these guidelines would not only ensure multidimensionality of pain assessment but also facilitate pooling of data for comparison.

Finally, the existing data are insufficient for analysis of pain experiences by OI type. Of the 371 OI participants captured in this review, at least 29.1% (*n* = 108) are confirmed to be diagnosed with OI Type I and 35.3% (*n* = 131) had an unspecified or unknown OI type. Future research requires the delineation of OI types permitting greater detail of pain assessments by OI type and identification of similarities and differences in pain experiences.

## Conclusion

In conclusion, this review highlights the limited attention given to the pain experiences of adults diagnosed with OI. As children, patients with OI can experience a larger number of fractures and bone malformations as they grow that can contribute to their pain experiences. During adulthood, bone growth and the number of fractures decreases, but acute and chronic pain still exists and presents similar problems to daily activities of living. Pain is a long-term symptom of OI requiring further research to better understand and manage pain in adults with OI.

## Supplementary Material

Supplemental Material

Supplemental Material

## supplementary material

Supplemental data for this article can be access on the publisher’s website.
