# Supplementary material for: Pain experiences of adults with osteogenesis imperfecta: An integrative review
Source: Can J Pain. 2018 Jan 30;2(1):9–20. doi: 10.1080/24740527.2017.1422115 (PMC8730592; doi:10.1080/24740527.2017.1422115)
Supplement: Supplemental Material [file UCJP_A_1422115_SM3850.docx]

Supplemental Table 2: Appraisal Scores

| **Studies appraised using the Quality Assessment Tool** | |
| --- | --- |
| Balkefors et al. (2013) | 46.4% |
| Bradbury et al. (2012) | 52.4% |
| Chevrel et al. (2006) | 45.2% |
| McAllion et al. (2002) | 33.3% |
| McKiernan et al. (2005) | 36.9% |
| Nicolaou et al. (2011) | 34.5% |
| Papagelopoulos et al. (1993) | 25.0% |
| **Studies appraised using the CAse REport (CARE) Tool** | |
| Furstenburg et al. (2010) | 60.0% |
| Hardenbrook & Lombardo (2006) | 46.7% |
| Iwamoto et al. (2003) | 66.7% |
| Iwamoto et al. (2004) | 63.3% |
| Khoury, Hamze & Laredo (2008) | 63.3% |
| Kim et al. (2013) | 40.0% |
| Rami et al. (2002) | 56.7% |
