# Supplementary material for: Pain experiences of adults with osteogenesis imperfecta: An integrative review
Source: Can J Pain. 2018 Jan 30;2(1):9–20. doi: 10.1080/24740527.2017.1422115 (PMC8730592; doi:10.1080/24740527.2017.1422115)
Supplement: Supplemental Material [file UCJP_A_1422115_SM3849.docx]

Supplemental Table 1: Database Search Strategy

| Medline, EMBASE,  Joanna Briggs | 1. Osteogenesis imperfecta.mp. or exp osteogenesis imperfecta/ |
| --- | --- |
|  | 2. brittle bone*.mp. |
|  | 3. lobstein*.mp. |
|  | 4. 1 or 2 or 3 |
|  | 5. exp pain severity/ or exp pain parameters/ or pain assessment/ or exp pain threshold/ or exp pain measurement/ or exp pain/ or exp pain intensity/ |
|  | 6. nocicept*.mp. |
|  | 7. nociception/ |
|  | 8. 5 or 6 or 7 |
|  | 9. 4 and 8 |
| CINAHL | 1. (MH "Osteogenesis Imperfecta") or "osteogenesis imperfecta |
|  | 2. brittle bone* or lobstein* |
|  | 3. 1 or 2 |
|  | 4. (MH "Pain+") or (MH "Pain Measurement") OR (MH "Pain Threshold") |
|  | 5. pain* or nocicept* |
|  | 6. 4 or 5 |
|  | 7. 3 and 6 |
